# Sustainable production of biofuels from the algae-derived biomass

**DOI:** 10.1007/s00449-022-02796-8

**Published:** 2022-11-04

**Authors:** Tehreem Mahmood, Nazim Hussain, Areej Shahbaz, Sikandar I. Mulla, Hafiz M.N. Iqbal, Muhammad Bilal

**Affiliations:** 1 Department of Biotechnology, Quaid-i-Azam University, Islamabad, 45320 Pakistan; 2 Center for Applied Molecular Biology (CAMB), University of the Punjab, Lahore, Pakistan; 3 Department of Biochemistry, School of Allied Health Sciences, REVA University, Bangalore, 560064 India; 4 Tecnologico de Monterrey, School of Engineering and Sciences, 64849 Monterrey, Mexico; 5 Faculty of Chemical Technology, Institute of Chemical Technology and Engineering, Poznan University of Technology, Berdychowo 4, 60695 Poznan, Poland

**Keywords:** Algae, Alternative fuels, Algal biomass, Sustainable energy, Cultivation techniques, Photobioreactors, Biomass conversion, Circular bioeconomy

## Abstract

The worldwide fossil fuel reserves are rapidly and continually being depleted as a result of the rapid increase in global population and rising energy sector needs. Fossil fuels should not be used carelessly since they produce greenhouse gases, air pollution, and global warming, which leads to ecological imbalance and health risks. The study aims to discuss the alternative renewable energy source that is necessary to meet the needs of the global energy industry in the future. Both microalgae and macroalgae have great potential for several industrial applications. Algae-based biofuels can surmount the inadequacies presented by conventional fuels, thereby reducing the ‘food versus fuel’ debate. Cultivation of algae can be performed in all three systems; closed, open, and hybrid frameworks from which algal biomass is harvested, treated and converted into the desired biofuels. Among these, closed photobioreactors are considered the most efficient system for the cultivation of algae. Different types of closed systems can be employed for the cultivation of algae such as stirred tank photobioreactor, flat panel photobioreactor, vertical column photobioreactor, bubble column photobioreactor, and horizontal tubular photobioreactor. The type of cultivation system along with various factors, such as light, temperature, nutrients, carbon dioxide, and pH affect the yield of algal biomass and hence the biofuel production. Algae-based biofuels present numerous benefits in terms of economic growth. Developing a biofuel industry based on algal cultivation can provide us with a lot of socio-economic advantages contributing to a publicly maintainable result. This article outlines the third-generation biofuels, how they are cultivated in different systems, different influencing factors, and the technologies for the conversion of biomass. The benefits provided by these new generation biofuels are also discussed. The development of algae-based biofuel would not only change environmental pollution control but also benefit producers' economic and social advancement.

**Graphical abstract:**

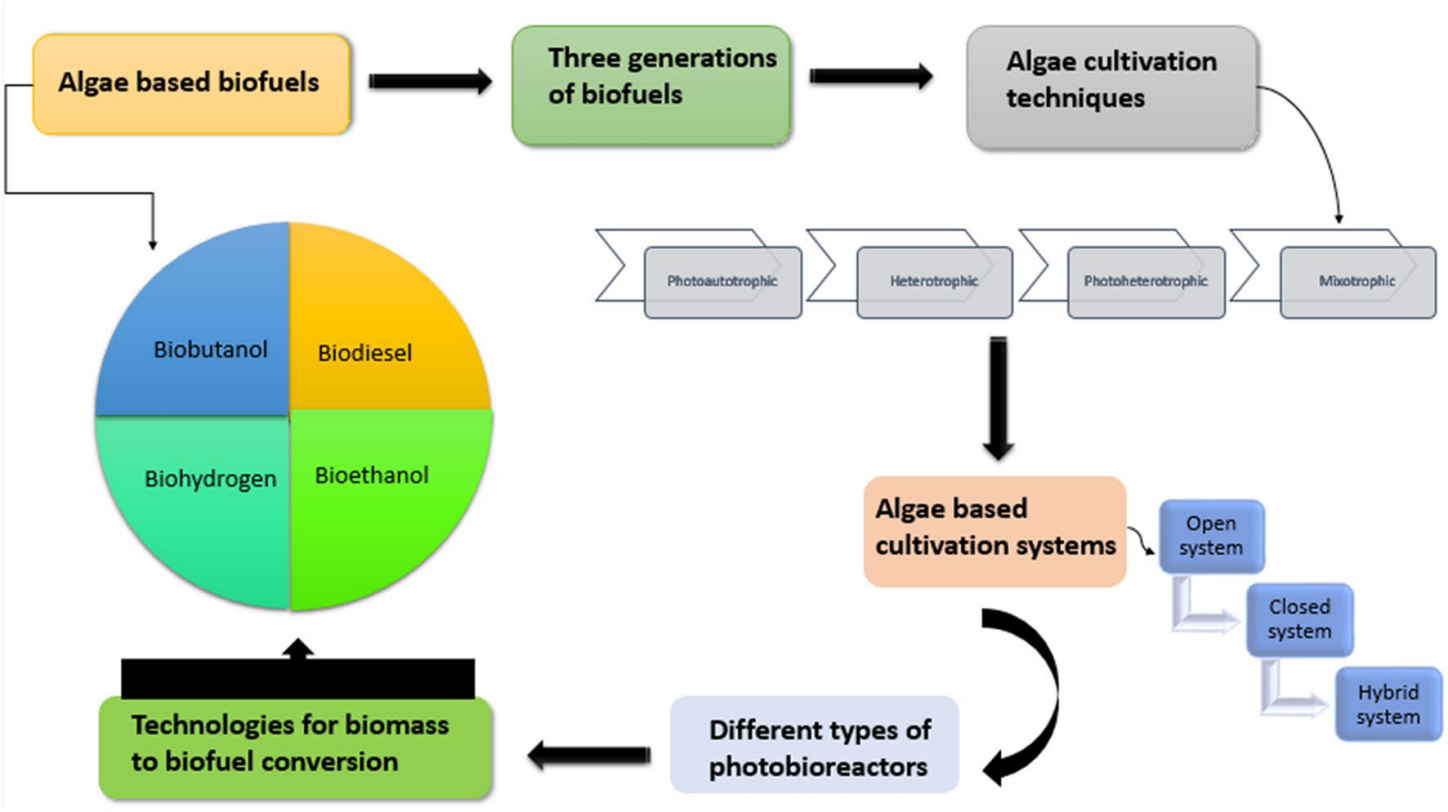

## Introduction

Commonly it is assumed that algae are photosynthetic autotrophs that mostly live in water, evolve oxygen, and are either made up of single cells or live in colonies or filamentous forms [1]. Algae are comprised of a large number of photosynthetic living beings that mostly inhabit aquatic surroundings. According to the size and morphological characteristics, algal species are usually classified into macroalgae and microalgae. Macroalgae which are also called seaweeds are made up of a large number of cells and can be seen with a naked eye. As compared to macroalgae, microalgal species can only be visualized with the help of a microscope and are highly important in the field of micro nanomedicine [2]. Based on existing pigments, brown algae, blue–green algae, and red algae are the three classes of macroalgae [3]. Although blue–green algae and bacteria share some common structural characteristics, blue–green algae were placed in the algal class because of the presence of chlorophyll and correlated complexes [4]. One more class of algae comprises the red algae. Species that belong to this class of Rhodophyta are eukaryotes that contain chloroplasts and phycobilins [5].

Brown algae named brown seaweeds are typically large macroalgae and have the comparatively immense ability to convert photons as a result of which biomass can be synthesized much more quickly. Brown algae are given more attention for the development of maintainable biofuels because their efficiency is considerably higher in contrast to that of cyanobacteria or red algae [6]. Microalgae have also arisen as a probable feedstock for the production of biofuels because a large number of microalgal strains have the ability of lipid accumulation, with a higher growth rate of biomass and greater photosynthetic production as compared to their counterparts that exist on land [7].

Difficulty to sustain and persistent debilitating of non-sustainable petroleum derivatives gave rise to the significance of inexhaustible fuel sources [8] a worldwide temperature alteration further amounts to the difficulties previously confronted [9]. These days, to move in the promising direction developed and underdeveloped countries are thinking about environmentally friendly power sources [10]. Biofuel is referred to as any fuel that is obtained from biomass that is either a plant, algae, or animal manure [11]. Biofuels are accepted to be the most natural amicable energy source. Biomass got from trees, agro backwoods buildups, marine or land plants, grasses, and harvests is the adaptable and significant sustainable feedstock for the development of biofuels [12]. The utilization of biomass as fuel is one of a handful of genuine systems to decrease the effects that greenhouse gases are causing. Contrasted with petroleum products, biomass ignition fundamentally diminishes CO_2_ and CO_2_ outflows and essentially lessens the debris obtained after burning [13]. As the conventional fuel resources are being depleted at a high rate, there is more focus towards the employment of alternative sources.

More than 50 years ago, the concept of employing algae as a source of food, feed, and energy was first proposed. During the energy crisis of the 1970s, when programs were started to manufacture gaseous fuels (hydrogen and methane), the production of methane gas from algae received a significant boost [14]. Our knowledge of cultivating algae for fuel has greatly benefited from the researchers' work on open pond algae growth [15]. The effects of various nutrient and CO_2_ concentrations were documented, the engineering difficulties of mass-producing algae were addressed, and a strong basis for algae-fuel research was established through the isolation and testing of thousands of distinct species. But in 1995, US Department of Energy’s (DOE) Office decided to end the initiative due to budgetary restrictions and low oil prices. Everything has altered in recent years. Concerns about "peak oil", the rising effects of atmospheric CO_2_, the United States' increasing reliance on fuel imports, and the associated hazards to energy security have all contributed to a resurgence in interest in biofuels in general and algae-based biofuels in particular [16]. Advantages in biotechnology have opened up new possibilities that were not possible during the years of former research, such as the ability to genetically modify algae to produce more oils and convert solar energy more effectively. The United States has seen the majority of activity in algae research and commercial production. Algae biofuels are currently being explored globally in both established and developing countries in Europe, Asia, and other regions. The US-based Algal Biomass Organization serves as the industry's chief spokesperson and a resource for data on the businesses pioneering the technology [17]. This article outlines the third-generation biofuels, how they are cultivated in different systems, different influencing factors, and the technologies for the conversion of biomass. The benefits provided by these new generation biofuels are also discussed. Given these factors, it is essential to remove the current bottlenecks to use microalgae for commercial purposes.

## Biofuel generations

The only renewable energy sources that can directly replace fossil fuels for current and future energy shortages are biofuel and biomass. These sources are environmentally favorable and renewable [18]. Generally, there are three generations of biofuels categorized based on their sources. Biofuels which are obtained directly from the food source are termed as first generation biofuels such as those that have been manufactured from the biomass comprising sugar, starch, and vegetable fats and oils [19]. The biofuels which fall under the category of the second generation are those that are produced from the plant biomass, which is mostly comprised of lignocellulosic materials, as this builds up most of the economical and ample nonfood compounds accessible from plants. But, in the present situation it is not economical to produce these fuels as there is a large number of mechanical obstacles that need to be avoided before their perspectives can be considered [20]. Second-generation biofuels, for instance, ethanol and methanol created from woody biomass, are more energy productive and more adaptable concerning their feedstock. The likelihood to utilize cellulosic and heterogeneous biomass recommends lower costs [21]. In any case, the ecological effect raised from biofuel combustion extraordinarily affects the carbon cycle (carbon balance), which is connected with the ignition of petroleum derivatives. Furthermore, the weariness of various existing biomass without suitable compensation brought about colossal biomass shortage, arising ecological issues like deforestation and biodiversity loss [22–24]. In an inquiry for feasible and practical options in contrast to non-renewable energy sources, past investigations have detailed the predominant abilities of green algae inferred biomass for the development of a better form: the third-age biofuels [25].

## Algal biofuels

**Fig. 1:**
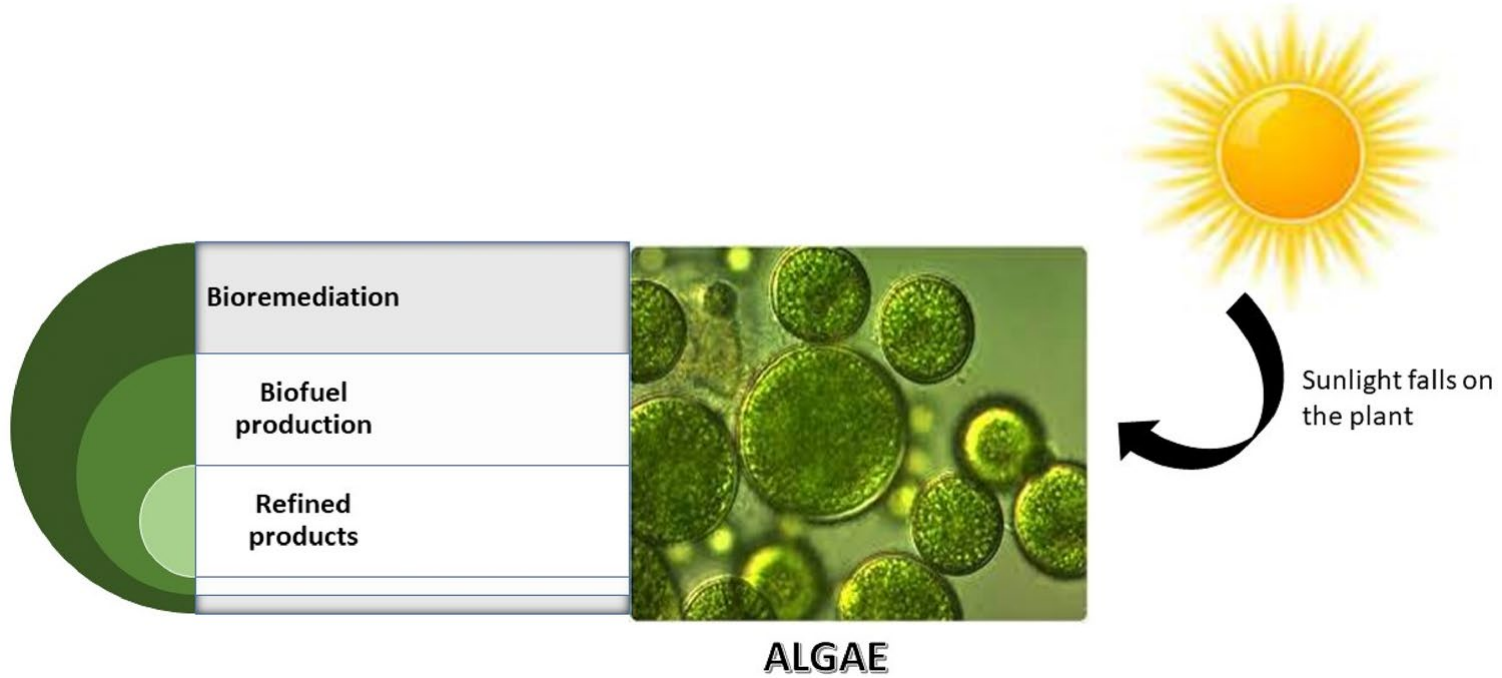
Cultivation of algae for biofuel production

Algae-derived biofuels are progressed sustainable fuels obtained from algal feedstock utilizing different conversion systems. This is because of the oil-rich arrangement of this feedstock that can be related to its capacity to plentifully photosynthesize [26]. Lipids, polysaccharides, unsaturated fats, pigmentary compounds, cancer prevention agents, and minerals are among the naturally dynamic mixtures found in algal concentrates (Fig. 1). By way of levels of oils among 20 and 50%, such as *Chlorella* sp., *Tetraselmis* sp.,* Dunaliella* sp.,* Isochrysis* sp.,* Nannochloris* sp., and *Nannochloropsis* sp., greater developments are reported. For the manufacture of biofuels, it is essential to produce lipids at high growth rates since high biomass productivity increases yield per harvest volume and high lipid content lowers the cost of extraction per unit product. Therefore, metabolic engineering of microalgae is required to enable the constitutive production of large amounts of lipids without compromising growth [27]. Moreover, lipids encompass the fatty acids which are essential for certain biofuels production [28]. Table 1 represents the lipid content of different algae species [29]. Notwithstanding biofuels, algae have been viewed as expected makers of synthetics that protect against viral, bacterial, and fungal infections and are also used for the production of antioxidants [30]. In a few viewpoints, microalgae feedstock is desirable to produce biofuels as microalgae does not need cultivable land and new water for development, they are not eatable hence no impact on food chain, can be developed to a few overlays regardless of occasional circumstances, alleviation of barometrical CO_2_ and waste water treatment [31].

**Table 1:** Lipid content and fatty acid content of different algal strains

Classes	Species	Lipid content (% dry weight)	Eicosapentaenoic acid (EPA) content (molar percentage)	Docosahexaenoic acid (DHA) content (molar percentage)	References
*Rhodophyceae*	*Porphyridium cruentum*	10–15	21	< 1	López Alonso et al. [159]
*Bacillariophyceae*	*Thalassiosira pseudonana*	24	15	1	Brown et al. [160], Bigogno et al. [161] and Pratoomyot et al. [162]
	*Skeletonema costatum*	13	10–20	1–5	Brown et al. [163] and Guihéneuf et al. [164]
	*Nitzschia *sp.	45–47	25–30	< 1	Budge and Parrish [165] and Pratoomyot et al. [162]
	*Odontella aurita*	7–13	> 25	1–2	Braud [166]
*Dinophyceae*	*Crypthecodinium cohnii*	20	45	< 1	Jiang et al. [167]
*Chlorophyceae*	*Tetraselmis suecica*	15–23	1–5	< 1	Brown et al. [163], Pratoomyot et al. [162] and [39]
	*Chlorella* sp.	28–32	1–5	< 1	Brown et al. [163], Pratoomyot et al. [162] and [39]
	*Dunaliella primolecta*	23	< 1	< 1	Brown et al. [163] and [39]
*Prymnesiophyceae*	*Pavlova lutheri*	20–25	> 20	10–20	Brown et al. [163] and Reitan et al. [168]
	*Isochrysis* sp.	25–33	< 1	10–20	Brown et al. [163] and López Alonso et al. [159]

Biodiesel, bioethanol, biohydrogen, and biobutanol are the basic biofuels derived from algal biomass.

### Biobutanol

Biologically prepared butanol is termed biobutanol which resembles gasoline and exhibits several promising applications. The most efficient method that has been used in past for the production of biobutanol is acetone–butanol ethanol (ABE) fermentation. Because of its extraordinary enactment and benefits, this valuable sustainable biofuel can be used along with the currently available fuels [32]. Because of the higher productivity rate and the presence of carbohydrates, microalgae are assumed to be the most promising feedstock for biofuel production. A large number of algal carbohydrates can be converted into simpler compounds, called monosaccharides, and then can be utilized in the fuel production process. Nowadays, biobutanol production is carried out by some of the world’s famous producers which include Gevo, Butamax, Green Biologics, and US Technology Corporation [33]. The production of butanol under the biological methods is done in the presence of an anaerobic environment and is considered a phase of ABE fermentation. For the very first time in 1862, Louis Pasteur was the first scientist who reported the microbial manufacturing of biobutanol [34]. Acetone–butanol–ethanol fermentation is a biphasic system in which during the acidogenesis phase, butyric acid and acetic acid are formed. After the production of acids, the re-assimilation of these acids results in the yielding of solvents including ethanol, butanol, and acetone [35]. However, this process of butanol production synthesizes the other solvents simultaneously, thus the selectivity rate of our desired product is decreased [36]. It has been described that with the inclusion of enzymes such as cellulases and xylanases, scientists engaged *C. saccharoperbutylacetonicum* to use algal biomass, which was obtained from the wastewater for the production of biobutanol. Green seaweed has also been employed for biobutanol production using the strains of strains *C. acetobutylicum* and *C. beijerinckii* along with the metabolization of xylose and glucose. Macroalgae obtained from a marine ecosystem have also been shown to be a promising candidate for butanol production such as Ceylon moss, a marine macroalgae feedstock was used and Clostridial strains were employed for the extraction of biobutanol from it [37].

### Biodiesel

Biodiesel refers to the biofuel which is comprised of mono-alkyl esters. These esters are obtained from organic oils, algae, plants, or animals by the method of transesterification [38]. The process of transesterification is an equilibrium method that employs the presence of a catalyst and processes the algae oil into biodiesel in the presence of potassium hydroxide-like alkali [39]. To produce the algae-derived biodiesel, a huge amount of algal biomass is required. Algae that are typically employed in biodiesel production are unicellular ones that are mostly found in the aquatic environment. These algal strains are usually characterized as eukaryotes that have immense potential to photosynthesize and have higher rates of growth and greater density of population. In the presence of optimal conditions, even in less than 24 h, a green alga has the potential of doubling its biomass [40]. For effective production of biodiesel, the algal strains need to be effectively cultivated and then the biomass is harvested from the reactor. The most important methods that are currently under use for microalgal harvesting include sedimentation, flocculation, filtration, electrophoresis, and centrifugation [41]. After harvesting, normally the dry weight of the biomass needs to be increased but if the aim is to develop the production system for biodiesel or biogas, then the necessitation of this step can be removed as the production of biodiesel and biogas can accept the moisture content of high amount and we can easily proceed the process directly after the wet extraction of lipids [42].

The US Department of Energy's Aquatic Species Program concentrated on the production of biodiesel from microalgae and provided the final report according to which, it was suggested that biodiesel could be the only feasible approach to provide us with sufficient fuel to substitute for the existing world utilization of biodiesel [43]. It has been estimated that if we use biodiesel which is obtained from algae as a substitute for the 1.1bn tons of conventional diesel which is globally produced per year then it would require only 57.3 million hectares of land, which would be immensely promising in contrast to other biofuels [44]. Although biodiesel has great potential, it cannot easily compete with other petroleum fuels due to certain limitations. Its high cost and the requirement for a huge supply of organic oils is a big hurdle in the competition. It has been predicted that when the cost of petroleum fuels will become high and when the supplies will diminish gradually, only then, the alternative biofuels will become more approachable to investors and purchasers [45]. For biodiesel to turn out to be the substitute fuel of choice, it necessitates a vast amount of inexpensive biomass. Utilizing novel and advanced cultivation methods, algae might permit the production of biodiesel to accomplish the rate and scale of manufacture required to race with, or even substitute, petroleum fuels [45].

### Biohydrogen

A diverse range of biochemical reactions derived from the microbes leads to the production of biohydrogen as a by-product. Biohydrogen exhibits a regular and short-lived nature. Biohydrogen can be referred to as the production of hydrogen by two mechanisms; thermochemical treatment or employing biological methods [46]. Biohydrogen is considered one of the most capable sustainable energy sources and can decrease the pressure which is being caused by a very limited supply of other resources. Moreover, it promotes the usage of the environmentally approachable technique. Biophotolysis of water, photo fermentation by photosynthetic bacteria, and anaerobic fermentation of biomass are some of the methods for producing biohydrogen [47]. A promising method of biohydrogen production is the biophotolysis of water using microalgae [48]. Another method of developing sustainable fuel production is the anaerobic fermentation of carbohydrate-rich biomass. Carbohydrates are efficient compounds where monomers are obtained and are employed in biohydrogen production. One such example of a monosaccharide is mannitol which is the probable substrate to produce biohydrogen using macroalgae [49]. Macroalgae is considered the most significant feed of biomass for biohydrogen production [50].

The process of direct photolysis has only been described in the microalgae species. This method employs the ability of green algae or cyanobacteria to photosynthesize. The process follows by the absorption of light leading to the splitting of water. The resulting electrons are then get transferred to enzymes such as nitrogenase or hydrogenase which will lead to the production of biohydrogen [51]. Indirect photolysis is a two-step method. In the initial step, highly photosynthetic biomass is prepared and in the next step, the process is followed by the anaerobic dark fermentation for the production of hydrogen. The step during which hydrogen is released is highly sensitive to oxygen. This is the reason the evolution steps of oxygen and hydrogen need to be separated in minimum time. Multiple models have been established for the process of indirect photolysis to be carried out. Most of these systems use algae and aim to use their capacity to photoautotrophically produce a huge amount of biomass per surface area [52].

As monomers are greatly employed in biohydrogen production, so the conversion of polymeric carbohydrates into monomers is considered a limiting step of the production process. To enhance hydrogen production using algae, a diverse range of pretreatments are employed to carry out the de-polymerization of polymeric sugars. The process of dark fermentation results in the negative net energy balance of differences between the energy which is evolved as hydrogen and the one that is used to produce biohydrogen. To make this whole procedure an economical one, the process of algal dark fermentation must be incorporated with a biorefinery method, where the outlets are commercialized into valuable biomolecules [53].

As it is known that the activity of hydrogenase is strongly inhibited by the presence of oxygen, researchers have utilized several methods to avoid this inhibition by avoiding the evolution of oxygen in the photosystem. One such method is to regulate the oxygen release by the usage of butyric acids and acetic acids [54]. Another limitation in commercializing the practical process is the higher costs of PBRs and the photon conversion efficiencies [55].

A more theoretical method needs to be developed to overcome the challenges that oxygen sensitivity is causing to encourage the studies and research on functional systems based on algae for producing biohydrogen. To improve hydrogen production, novel techniques must be introduced for the separation of oxygen from other biochemical reactions. It has been suggested that using the genetic engineering approach, such algal strains can be developed that can withstand and easily tolerate oxygen. The forthcoming role of biohydrogen as an unpolluted energy source for fuel cells manufacturing approximately zero emanations, and as a transitional energy carrier for storing and transporting sustainable energy, is progressively renowned globally [56].

### Bioethanol

In the present world, huge attention is being given to bioethanol due to its ecological advantages. Bioethanol can be obtained by all three generations of feedstocks including plants, lignocellulosic, and algal biomass [57]. To make bioethanol using algal biomass, the cultivated algae are harvested and then dried to remove nearly 50 percent of the moisture so that a solid material can be obtained and handled with ease. For this purpose, the harvested biomass of algae is made to undergo an appropriate process of dehydration to reduce the quantity of water before the oil is extracted. The moisture content is normally removed by a feasible drying procedure. Algal biomass can be dried by various approaches such as freeze drying, sun drying, or spring drying [58–60]. The process which is typically employed for the production of bioethanol from the algal biomass is fermentation. This process is used for the conversion of starch, sugars, or cellulose existing in algal biomass into bioethanol. This process is carried out by crushing biomass followed by the transformation of starch into sugars. Water and yeast are then mixed with it in the bioreactors which are called fermenters [61]. Yeast is used because it is involved in the breaking of sugar and transformation into bioethanol. Distillation is then carried out as a cleansing method for the removal of water and other contaminants in the thinned alcohol leading to the production of concentrated ethanol. The desired concentrated bioethanol is drained and converted into fluid form. This bioethanol is used for the supplementation or substitution of fuel in cars [62]. The production of bioethanol using microalgae can be used to surmount those environmental issues problems where producing bioethanol from conventional feedstock is found to be emitting more greenhouse gases than fossil fuels as a consequence of the feedstock manufacture and applications throughout the procedure [63].

## Algal cultivation techniques

In addition to nutrients such as phosphorus, nitrogen, potassium, zinc, and calcium, algal cultivation necessitates water, carbon dioxide, and sunlight to yield biomass through photosynthesis, by the conversion of solar energy into chemical energy stored in the microalgal cells. Four major types of algal cultivation methods have been recognized based on certain conditions required for growth. These cultivation techniques are photoautotrophic, heterotrophic, photoheterotrophic, and mixotrophic cultures [64, 65]. Under the mixotrophic mode of cultivation, microalgae can drive both photoautotrophy and heterotrophy and can exploit equally the inorganic and organic sources of carbon [66]. Under photoautotrophic mode, chemical energy is made by the process of photosynthesis, during which processed microalgae exploit light as the energy source and inorganic carbon as the carbon source. A CO_2_-rich environment could enhance biomass productivity to a certain extent [67].

Photoheterotrophic cultivation which is also called photo-metabolism besides photo-assimilation is the mode of cultivation that requires light and this light is needed to utilize the organic compounds as a source of carbon [68, 69]. Heterotrophic cultivation exploits organic carbon materials as a source of energy and carbon to promote algal growth [70]. Among the above-mentioned approaches, photoautotrophic production is most extensively used because it is appropriate for large-scale algal biomass production [71]. The cultivation systems for algal production consist of three simple choices—open, closed, and hybrid systems. While the open systems are cost-effective and the closed systems are more efficient in nutrient removal, the hybrid systems are a culmination of open and closed systems specifically meant for high productivity in terms of biomass generation [72].

### Open pond systems

Around the globe, open pond systems with the usual depth of 1530 cm, are commonly used to cultivate algae. Carbon dioxide which is readily present in the atmosphere can be alleviated by algae. The most generally practiced schemes for research and industrialized algal cultivation include the raceway pond, the closed pond, the shallow big pond, and the circular pond tank [73]. The cultures that are developed in open ponds can stand protection from adversarial ecological circumstances (rainfall, temperature, and luminosity) by the usage of a greenhouse. Microalgae that cultivate in adverse conditions, such as a basic medium or highly saline one, should be approved in command to attain axenic cultures [74]. Open ponds built in a wastewater treatment plant can be circular or driven by gravity flow [72].

The location of a pond is the most basic criterion for open systems. The location should be chosen based on maximum sunlight provision and the availability of all the requirements needed by the algal strains. The stirring unit is mostly absent in these kinds of systems as a result of which there is poor mixing but overall, these culture systems allow the culture process to be handled and monitored most simply and economically. The natural pond is typically not as much of a half meter deep as a consequence of which light breaches the water and a large number of algal cells can absorb it. An earlier report suggested that plastic films can also be exploited by layering them over the water surface for improved temperature control. Several algal strains, mostly for example *Dunaliella salina*, can be cultivated in these types of open systems for profitable motives [75]. An elongated spinning arm is set in the center of the pond which actions like a clock dial and executes a function of a paddlewheel which is conversant in the structure to that of a raceway pond. Mixing of algae cells and culture media is extra effective as compared to an unstirred pool, but as the algae get exposed to the environment, the contamination becomes unavoidable. As per the research literature, the efficiencies in circular ponds range between 8.5 and 21 g/(m^2^ d) [76].

The raceway ponds for algal cultivation have been used since the 1950s. Initially, they were used for the *Spirulina* cultures. They can be consisting of either a racetrack channel or an oval channel. They are normally constructed utilizing a concrete solid [77]. Raceway ponds offer a continuous supply of nutrients and carbon dioxide along with the recirculation of algal culture. They are armed with a paddle wheel to be responsible for mild mixing to inhibit sedimentation. An aerator can be utilized for the intensification of air flow rate and hence carbon dioxide utilization [78].

A sustainable process involves using wastewater for algal production, which would provide the combined advantages of bioremediation of nutrients like phosphorus and nitrogen along with the production of biofuels. Considering brackish water, wastewater, and marine waters as a growth medium for algae can help to overcome important ecological challenges, but it will necessitate substantial research [79]. The productivity of algae in large-scale ponds is firmly governed by pH and dissolved oxygen concentration. Principle component analysis is a powerful instrument to perceive the restricted reduction in productivity made by unsuitable processing of the cultures [80]. To become a feasible option for scaling the production of algal biomass, open systems have to become cheaper to build and operate while sustaining robust and productive growth [81].

The efficiency of open ponds is questionable, even though their construction and operation costs are modest. An open system is difficult to monitor as there is a higher need for land, and there is a higher risk of contamination, as well as constraints due to weather and light intensity. The main drawbacks of an open-air system are its sensitivity to weather, season, and time of day. Some drawbacks of open pond systems prevent their use such as the inability to provide us with monocultures because several native algae and algae graze contribute to contamination. Pond temperature is not usually under control and the intensity of light is reliant on arriving solar insolation. Hence, the effectiveness of the open ponds is reliant on the native daytime deviations in temperature and solar insolation. While the cooling generated from the evaporative process somewhat controls the temperatures of open ponds, it as well points to a substantial loss of water [82, 83]

### Photobioreactors

#### Horizontal tubular photobioreactor

At the commercial level, algal growth is widely done using horizontal tubular photobioreactors (HTB). This kind of bioreactor consists of a long arrangement of tubes that can be made of either transparent silicone, glass, or plastic material. These tubes are placed horizontally, and their diameter is kept small so that the area for the penetration of light is enhanced. This large area for illumination makes the tubular photobioreactor a good choice for the cultivation of algae. The circulation of algal cells in tubular PBR can be done by airlift technology quantum fracturing or using a centrifugal pump. Contemporary methods have been discovered in scheming tubular PBRs to guarantee a thin layer of culture suspension that is free from contamination along with extraordinary exposure to light and decreased energy requirement. However, the rise in the diameter of the tube can lead to a decreased surface-area-to-volume ratio hence less illumination. The increased diameter of the tube can also lead to the unequal distribution of solar energy to algal cells present at different levels in the tube. The longer tubes can cause the accumulation of oxygen which plays an inhibitory role in the photosynthesis of algal strains. These difficulties can limit the scale-up of the tubular photobioreactor. Another limitation of tubular photobioreactor is that temperature control in tubular PBR is not an easy task. Although, thermostats and cooling tubes can be used they are quite expensive to install. HTB can be scaled up by placing the tubes either above one another or using the coiled tubes. The rise of pH of the cultures in these kinds of bioreactors requires recurrent carbonation as a result of which the cost of algal production would be increased. It also requires a large land area to be operational as compared to the vertical ones [84]. Recently a new HTB which is named ‘Biocoil’ has been designed in the UK. The material that is exploited for its manufacturing is Teflon or low-density polyethylene and tested effectively at an experimental scale (2000 L) for growing several strains of algae. The poor gas exchange and the large gas gradient along the tubes, which is brought on by the majority of the gas exchange taking place in a separate chamber, are drawbacks of employing these types of reactors. High energy input and occasionally an accumulation of biomass in the tubes are additional drawbacks [85].

#### Vertical column photobioreactor

This kind of bioreactor is made up of glass or acrylic tubing which is placed vertically and allows the light to penetrate them. A gas sparger system is used for introducing the tiny bubbles of inlet gas into the reactor and allows the efficient mixing, mass transfer of carbon dioxide, and removal of oxygen. Usually, there is no incorporation of a physical agitation system in a vertical column photobioreactor. Vertical PBRs can be classified into airlift reactors and bubble columns based on arrays of liquid flow [86].

#### Bubble column reactors

The height of a bubble column reactor is larger than twice that of the vessel's diameter. These are cost-effective and are made of a large surface area for illumination. No moving parts are required in these kinds of reactors as efficient mixing and mass transfer are carried out using a sparger. The design of the sparger plays a key role in the enactment of the photobioreactor. Perforated plates are normally utilized as spargers for shattering and redistribution of the coalesced bubbles. Light is provided by an external source. By moving from the central dark zone to the upper light zone, this liquid circulation develops a differential gas flow rate which is crucial for photosynthetic efficiency. Bubble size, though, seemingly is as well vital for diminishing shear damage to cells [87]. Because of the high mass transfer, low energy costs, and exceptionally low physical stress, some bubble column PBRs are armed with a rubber membrane diffuser or double spargers to expand the mass transfer of gases: availability of carbon dioxide and removal of oxygen. If the dual spargers are used, the efficacy of CO_2_ transfer is amplified fivefold compared to that of conventional sparging. According to the membrane diffuser's performance, the membrane's slits are more like holes with elastic lids that serve as valves to stop bubbles from entering the gas stream. Thus, the membrane diffuser serves as a one-way valve to stop liquid backflow [88].

Airlift reactors consist of a vessel having two interrelating zones. The gas blend streams up to the surface from the sparger in one cylinder, called the gas riser. The further area named the down comer, is the place where the medium streams down in the direction of the base and flows inside the riser and the down comer. The time gas residence in diverse zones impacts gas–liquid mass transfer, heat transfer, mixing, and turbulence and, therefore, is important to control the operation. A rectangular airlift photobioreactor has presented improved mixing features and improved photosynthetic competence, though its design is intricate and, therefore, is not easy to scale-up. The main limitation of using these kinds of vertical bubble column PBRs is that some of the algal strains such as *S. costatum* and *C. muelleri * have been reported to experience sheer stress in algal tubes and because of the pressure provided by the pumps, some algal cells cannot survive [89]. Recently, the effectiveness of a unique zigzag-flow column photobioreactor (ZZ-flow PBR) created to cultivate *A. platensis* with high biomass has been assessed. Four optimized zigzag baffle structures were put over the outer (riser) segment of the ZZ-flow PBR during installation. In comparison to traditional column PBR, the rate of intracellular photosynthesis and electron transport was improved, increasing biomass output and CO_2_ fixation [90].

#### Helical type photobioreactor

Helical PBRs are made up of a coiled transparent and flexible tube with a tiny diameter and a degassing unit that can be both detached and joined. The culture is driven over a lengthy tube to the degassing device using a centrifugal pump. The energy needed by the centrifugal pump recirculating the culture and accompanying shear stress bounds the marketable application of this type of photobioreactor, which may be scaled up by simply adding a light-harvesting device. Another downside of the technology is polluting the inside of the reactor [91]. The helical PBR was also given a cone shape with a cone angle of 60°, resulting in a conical helical reactor. For the conical helical system, the angle and height are carefully determined. Polyvinyl chloride tubing was coiled in a conical framework to create the conical helical reactor. The liquid was recirculated using an air pump. This system also includes a degassing system and a heat exchanger for temperature regulation. The photoreceiving area and thus photosynthetic productivities rise by a factor of two when employing a 60°. Among all other cone angles examined for this reactor, the photosynthetic efficiency of 6.84 percent was the highest. The key benefit of the cone shape is the increased light-harvesting efficiency while maintaining the same basal area. Another benefit of this type of reactor is that it requires less energy and places less mechanical stress on algae cells. Because of its defined angle and size, increasing the number of light collecting units is the only method to scale-up, but it results in more energy loss in the intricate branches of flow networks [92]. Low gas exchange, high shear stress, the buildup of biomass in the tubes, and the high energy input are all drawbacks of this type of reactor [85].

#### Flat-panel PBR

A flat panel photobioreactor usually consists of a transparent vessel that is made up of glass, plexiglass, or polyethylene film, and its thickness lies between 56 cm. The surface-area-to-volume ratio of these bioreactors is greater as compared to the tubular bioreactors. How flat-panel PBRs are designed entails suitable alignment to capture the solar potential for algal growth. The panels are ordered in head-to-head or parallel plates to prevent self-shading which is the main cause of photosynthetic inhibition leading to reduced algal growth. The provision of light might be achieved using light-emitting diode lights or optical fibers that achieve effective radiance to encourage the thriving growth of algae. Water is sprayed over the surface of panels for controlling the temperature. Heat exchangers are also used for this purpose, but they are not as cost-effective. Flat panel bioreactors frameworks when working at indoor surroundings, the elements, for example, space among light sources and panels, temperature impacts, enlightenment of one or both panel sides, light way are pivotal. Expanding volume results in the expansion of hydrostatic tension, in this way making scale-up troublesome. In addition, the hydrodynamic pressure might influence microalgae development.

In the aim to mass cultivate green algae, the flat panel PBR is incredibly suggested attributable to an extraordinary proficiency of photosynthesis and lower measure of disintegrated oxygen amassing, however, there are challenges connected with sterilization. However, these kinds of PBRs are exceptionally useful but the difficulty of scaling up and high operating costs sometimes limit the use of flat panel PBR. A less expensive design of flat panel PBR has been suggested where they utilized plastic packs inside the rectangular casing [93]. However, flat plate systems may also suffer from certain limitations. For example, few glitches such as greater space necessitation, a huge amount of solar energy, chances of hydrodynamic stress to some algal strains, problematic maintenance, low effectiveness in terms of mass production per unit of space, and fouling up of the channels [94].

#### Stirred tank photobioreactor (STR)

The most convenient type of reactor is the stirred tank reactor, which uses impellers of various sizes and shapes to generate mechanical agitation. Baffles are used to minimize the vortex. At the bottom, CO_2_-enriched air is bubbled for the provision of a carbon source for algae development. This sort of bioreactor ought to be converted to a photobioreactor by externally illuminating it with fluorescent lights or optical fibers. The unemployed sparged gas and created oxygen throughout photosynthesis are separated from the gassed liquid to the gas phase by a large disengagement zone. Stirred tank reactors were first presented as a way to produce microalgae photo autotrophically utilizing artificial light or sunlight since they were an industry and laboratory standard. To develop *Selenastrum capricornutum*, a Hydraulically Integrated Serial Turbidostat Algal Reactor (HISTAR) system with an entire volume of 3.6 cubic meters was utilized. Two sealed turbidostats and a sequence of open hydraulically coupled continuous flow stirred-tank reactors comprised HISTAR (CFSTRs). The inoculated culture's biomass was amplified by the CFSTRs. This technology has been lately exploited in the direction of creating a deterministic system to anticipate microalgal yield to determine practical viability for far-reaching application; though, no reports of future deployment have been made [95]. However, the chief drawback of this system is the small surface-area-to-volume ratio that reduces light collecting effectiveness. The usage of optical fibers for illumination has also been attempted, although this has the issue of obstructing the mixing pattern [96].

### Advanced systems

The hybrid frameworks are exceptionally planned frameworks which are a summit of the two sorts of development framework, conservative being used and implied for huge scope green growth development. These frameworks defeat the constraints of open ponds and the high starting working expense related to closed systems. For this situation, algal growths are first refined in a PBR, to accomplish high-density inoculants and afterward moved to an open framework, accordingly, working with the fulfillment of ideal biomass creation. The possibilities of defilement in open frameworks are significantly diminished when moved to the open framework, with algae getting predominant and contending successfully with different microorganisms [73]. Hybrid systems, on the other hand, involve substantial infrastructure, costly maintenance, and ongoing supervision [97].

A modified cultivation technique known as an Algal Turf Scrubber (ATS) was first presented by Professor Walter Adey at the beginning of the 1980s [98]. A downward-sloping surface that allows water or influent to flow across it intermittently or continuously is provided by an ATS culture system, which encourages the growth of macroalgae [99]. Microalgae in the ATS system grow effectively as a result of proper inorganic compound intake and photosynthesis-based dissolved oxygen release. The procedure has some downsides, including the need for enough acreage, a lesser capacity for processing wastewater, and significant infrastructure [97].

## Technologies for biomass to biofuel conversion

**Fig. 2:**
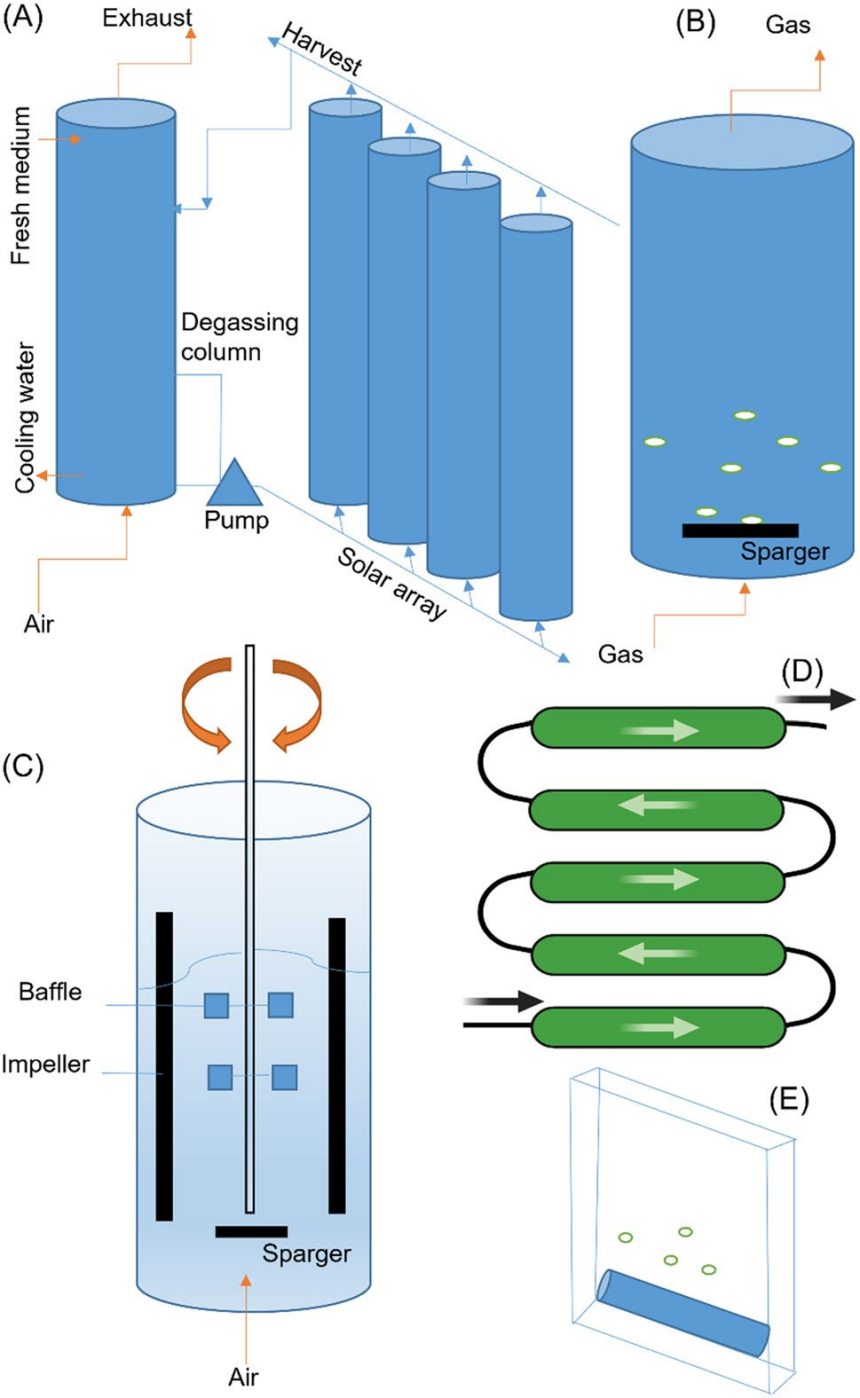
Different configurations for photobioreactors: **A** tubular photobioreactor, **B** bubble column photobioreactor, **C** flat panel photobioreactor, **D** helical tubular photobioreactor, **E** a simply stirred tank photobioreactor

Available processes that carry out the transformation of algae-derived biomass to diverse energy resources are categorized as thermochemical conversion, biochemical conversion, chemical pathways, and direct combustion (Fig. 2). Algal biomass can be converted into biofuels with the help of thermochemical methods including pyrolysis, gasification, and superficial liquid extraction, or by carrying out hydrothermal liquefaction. Apart from the sugars and lipids, all of the algae-derived biomass can be converted into biofuels using these techniques. After cultivation, if the further processing of algal biomass involves the use of the thermochemical method, there is no need of employing special surroundings such as nitrogen scarcity during the cultivation process in hope of achieving maximum content of lipid [100]. The process of gasification involves the algae-based biomass reaction in a gasifier under the partial oxidation of air. This process is carried out with any kind of combustion and in the presence of oxygen, air, or steam. A number of other downstream methods also accompany this traditional method. The gasification process along with some other downstream processing techniques eventually results in the production of carbon monoxide, methane, and hydrogen, combined with definite undesired by-products that are solid [101].

Another method of thermochemical conversion is pyrolysis during which thermal decomposition of algae is done without using any air or oxygen. The procedure is usually operated under atmospheric pressure and the temperature employed for heating usually ranges from 400 to 600 °C for conventional pyrolysis. However, 800 °C and a least 300 °C temperature are employed for microwave pyrolysis and catalytic pyrolysis, respectively [102]. One main advantage of pyrolysis is that Pyrolysis greater yields of bio-oil are conceivable (approximately 57.5% w/w). However, this process is limited by its need for dried biomass having least of the moisture content [9]**.** During the hydrothermal liquefaction, usually the algae make approximately five to fifty percent of the slurry feed. Extremely high temperatures nearly 250–500 °C to physically and chemically convert the biomass into fuels. Auto thermal water is used under 5–20 bar pressure and the process is carried out either in the presence or absence of a catalyst [101, 102]. Liquefaction results in the production of bio-oil along with some other by-products of methane that exists in a gaseous state. Although the reactions are highly complicated greater yields can be obtained using this reaction [9]. Microalgae can be converted in a biochemical process using either microbes or enzymes by the action of which algae are broken down into fuels. In contrast to the thermochemical conversion methods, biochemical conversions usually occur at a slower rate and are not much energy-intensive. Fermentation, photo-biological H2 production, and anaerobic digestion are some of the biochemical conversion strategies and repeatedly necessitate pretreatment of the biomass, particularly before carrying out the fermentation and anaerobic digestion [103].

Biomethane is generally produced by the anaerobic digestion during which carbon dioxide and traces of hydrogen sulfide are also manufactured along with biomethane from algal biomass from the enzyme or microbe catalyzed conversion of organic matter. Anaerobic digestion is usually appropriate for the organic matter that exhibits a large amount of moisture such as algal biomass. The three steps of this process are carried out in sequential order: hydrolysis, acetogenesis, and methanogenesis. Since hydrolysis acts as the rate-limiting step in the process of anaerobic digestion, the algal liability to the attack by an enzyme is a significant aspect that might be accomplished through pretreatment. Even though the typical power-driven pretreatment is the common custom designed for microalgae, they remain to be extra suitable to be used for thermal pretreatment [104]. Microalgae that are comprised of greater starch-based content are typically employed for the process of fermentation. This process involves the conversion of the principal components like sugars and starch into bioethanol by carrying out hydrolysis. The process is further followed by the fermentation by yeast leading to the production of bioethanol. The algal cell wall is then exposed to the pretreatment processes for the releasing of carbohydrates either by sonication or enzymatic mechanisms [105]. However, this process is limited to the transformation of lipids only and does not exploit starch and protein portions of feedstock [9].

## Factors affecting algal cultivation and biofuel production

Both biotic and abiotic factors may influence the biomass yield and thus the production of biofuels. Light, temperature, pH, nutrients, and carbon dioxide are among the abiotic factors while the specie of algae used for cultivation is a biotic factor that can have an impact on the yield and productivity.

### Light

It has been observed that the growth and the biomass accumulation of the algal species during cultivation are highly reliant on the wavelength as well as the intensity of light. In some cases, it has been shown that as the intensity of light is increased, the lipid content also increased [106]. It is known that the choice of algae as a feedstock is typically made by keeping in mind its ability to highly photosynthesize. As light is required for the process of photosynthesis and growth, it acts as one of the most important factors involved in controlling lipid accumulation and lipid enhancement. The shading effect of the light is also observed by some researchers leading to the understanding that the shedding light inhibits the growth of some algal strains and as soon as the shading light materials were removed, the agar continues to grow with ease and in a quicker manner [96]. It has been found that growth achieved using the fluorescent light source is comparatively enhanced as compared to the other sources of light [107].

### Temperature

Temperature is among several significant aspects that contribute to the growth of algae, lipid accumulation, and biofuel production. Most of the algae species grow optimally in the range of 20–35 degrees centigrade. But some species fall in the mesophilic category and approximately 40 degrees is still a bearable temperature for them. Overheating or heating at a temperature that is less than the required one can lead to the reduction of the yield and cell damage, respectively [108]. It has also been observed that the number of lipids in the algal biomass was shown to be decreased both by extremely high and extremely low temperatures [96].

### Carbon dioxide

Atmosphere, gas emissions from the exhausts of industries, and soluble carbonates are some of the important sources by which carbon dioxide can be obtained [109]. A greater amount of carbon dioxide is a necessity for the production of higher algal lipid content [110]. Different works of literature provide different evidence for the impact of carbon dioxide on lipid accumulation. It has been observed that the growth of algae is enhanced, and the process of fatty acid synthesis is diminished by decreasing the concentration of carbon dioxide, on the other hand, fatty acid synthesis is enhanced by a higher amount of carbon dioxide. However, increasing the concentration of carbon dioxide will highly affect the carbon chain. One of the studies done on the *Chlorella pyrenoidosa* SJTU-2 and *Scenedesmus obliquus* SJTU-3 has shown that at 10 percent of carbon dioxide, the growth was enhanced. However, the accumulation of lipids and the synthesis of fatty acids increased when 30–50% of CO_2_ was employed [111].

### pH

Lipid accumulation, oil synthesis, and the enzymatic activity for the growth of algae are highly influenced by pH which is among the most important factors. The growth of algae is enhanced by the environment which is acidic to some extent or has a neutral pH. But the nutrient medium has the presence of carbonic acids that can result in a lower pH as a consequence of which, the circumstances become opposing to the algal growth. Moreover, as the concentration of bicarbonates at low pH is less than that at high pH, this causes a negative influence on the carbon Integration for lipids formation [110, 112]. The change in pH has an impact on the microalgal biological reactions in a variable manner. It has been observed that the augmented pH eagerly repressed the cell division of *Chlorella*, activated the discharge of autospores, and as a final point give rise to TAG exploitation (Fig. 2) [113].

### Nutrients

If the limitations of various nutrients are assumed, then diversification of biochemical compounds can be detected in algae. However, it depends upon the type of nutrient which is limited and the extent of the limitation. If the pH and temperature are maintained at the optimal range, then the rate at which algae grows is directly proportional to the rate at which the extreme limiting nutrient is uptaken. The most vital macronutrients that are mandatory for the proper growth and development of algae are phosphate and nitrogen. Moreover, the nutrients that are non-minerals in nature and are essential for algal growth are carbon, oxygen, and hydrogen. However, the growth and metabolic pathways of algae are not challenged by the profusion of oxygen and hydrogen [9]. It has been shown by some scientists that the nutrients which are released from the algal biomass can be recycled and again used in the nutrient media. Such nutrients are first converted into a co-product that exists in the liquid state and is then made available to be used again. An example was shown by growing a bi-culture of two algal strains and recycling and using the nutrients that were released during the process of carbonization as the feedstock for the production of biodiesel [114].

### Algae species

The biochemical makeup of the algal community has an impact on its biomass's capacity for producing low-cost biofuels. The colonial species, which often dominate in high-rate algal ponds and have the advantage of being readily harvested by simple, low-cost gravity settling, have received little attention. Five wastewater colonial algae species that are frequently found in high-rate algal ponds were examined for their efficacy in wastewater treatment and their potential value for producing biofuels in terms of their biochemical composition and biomass energy yield: *Coelastrum *species, *Desmodesmus* species, *Pediastrum boryanum*, *Micractinium pusillum*, *Mucidosphaerium pulchellum*, and others. Summer has higher algal biomass output, lipid, and energy content than winter, depending on the species. Under both summer and winter circumstances, the *Mucidosphaerium pulchellum* and *Micractinium pusillum* cultures produced the most biomass and had the highest lipid and energy contents. *Micractinium pusillum*, however, settles far more readily than *Mucidosphaerium pulchellum*, indicating that of the colonial algae species examined, *Micractinium pusillum* offers the highest promise for both wastewater treatment and low-cost biofuel generation [115]. Although no polyculture is shown to produce more biomass than the most prolific monoculture, greater species diversity significantly can boost the output in comparison to the average across monocultures. However, there are data that suggest polycultures may be less likely to make undesirable crop function trade-offs, supporting the idea that variety can support several functions simultaneously [116].

## Limitations and benefits

In contrast to the first and second-era biofuels, algal biofuels do not compete much with fossil fuels [117]. Algae exhibit a considerable energy need of the numerous equipment and capital contributions of the augmented agronomy rotations in contrast to the terrestrial feedstock [118]. This is the reason for a comparatively lower return of net energy and diminished ability to compete [119, 120]. This significant need for energy can hypothetically lead to a loss of net energy for algae-based biodiesel, or at best a bordering improvement assumed the present expertise [121]. It is assumed that PBRs show greater costs for cultivation and eventually a decreased ratio of energy. One main disadvantage of using PBRs for algal cultivation is that the construction of photobioreactor and the need to circulate the culture demands for most of the energy cost [120, 122]. It has been discovered that key drivers of financial risk include both weather and pricing changes. This is the first probabilistic assessment of weather-related production consequences for algae growers, which is important given the industry's global expansion and the fact that the US 2018 Farm Bill now recognizes algae as a new commodity eligible for crop insurance [123].

Nonetheless, where the manufacturing industry is somewhat novel, there is the possibility of enhancements in the strains of algae and manufacturing tools that can guarantee an advanced likelihood of a net energy balance that will be positive, however, yet there is no certainty [124].

**Fig. 3:**
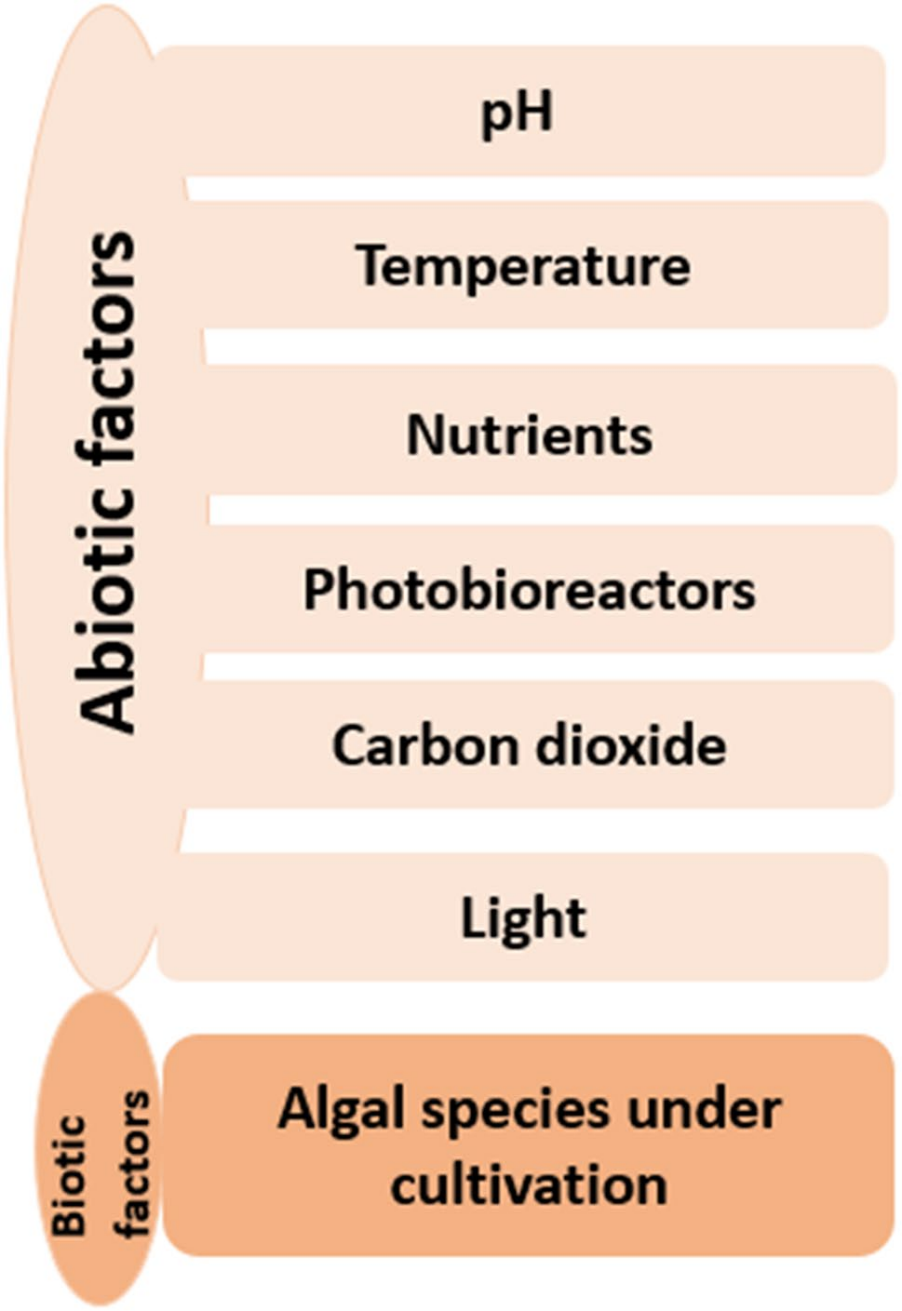
Factors affecting algal cultivation and biofuel production

Algae-derived biofuels might be feasible as probable air travel petroleum if assumed for their dense energy characteristics and also shows great potential in research for the companies associated with air travel [125]. Some of the advantages of using algae as a potential source of biofuel are also discussed in Fig. 3. A large number of by-products that can be extensively used on a commercial level can be prepared by microalgae [126]. The forthcoming marketable feasibility of microalgae as a feedstock for the production of biofuel may also be determined by a suitable commercial usage of these by-products [126, 127].

If open pond systems are compared with the PBRs, it is found that the PBRs exhibit a more effective ratio of energy [119]. Open-lakes were likewise shown to have a lesser amount of energy-demanding development, with more critical energy expenses being brought about by reaping and drying stages, tallying as much as multiple times the energy proportion [118, 127, 128].

**Fig. 4:**
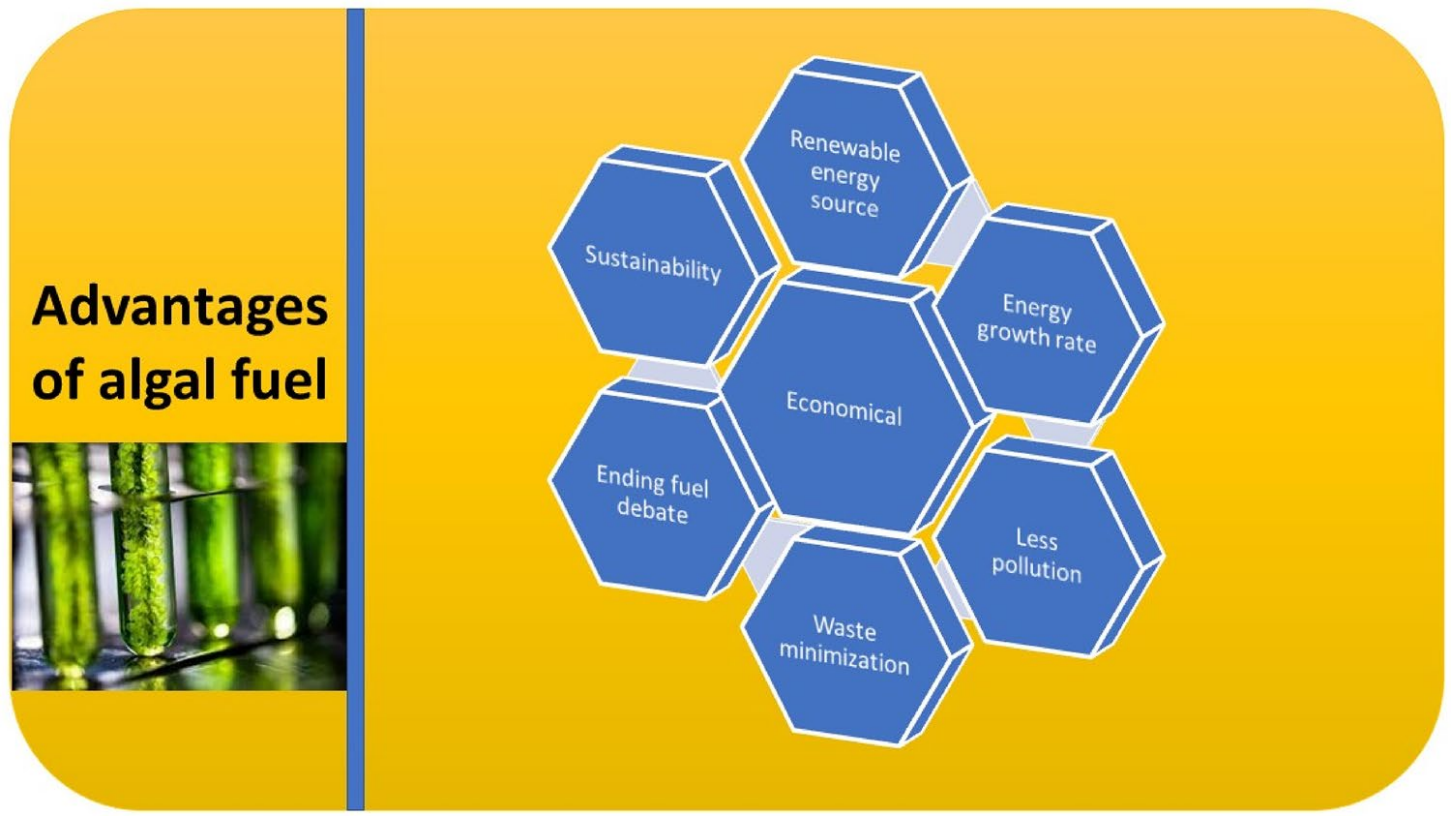
Advantages of algal fuel

Just like terrestrial cultivation, the biomass is prepared via photosynthesis with the help of algae [39]. Although this process occurs with more affectivity in algae regarding the farmed are but the conversion process is comparatively still not economical [129, 130]. In literature, there is a great emphasis on the importance of developing commercially feasible productivities to lesser net costs [131]. Production of microalgae at the commercial level is additionally expected to have positive net fossil fuel by-products, in contrast to its earthbound partners, because of the precise manufacturing climate and associated apparatus that need fossil-inferred power [128, 132]. Furthermore, the utilization of petroleum products in the downstream handling of the biomass can likewise check the greenhouse gas sequestration paybacks accomplished in the upstream development, as with regular biofuels (Fig. 4) [75, 133].

It has been suggested that during the process of cultivation, recycling the outlet gas can result in a net decrease in carbon emissions. Advantages of effective carbon fixation can also be achieved if the flue gas is introduced as carbon dioxide input into the medium for algal growth [134, 135]. The growth of biomass is not affected by this technique [124]. Some exploratory and applied research on the proficiency of a microalgal strain to utilize a highly concentrated vent gas supply exhibited the attainability and productivity of this application past earthbound horticulture [130, 136–138]. Notwithstanding this sequestration advantage, the net carbon dioxide help from microalgae is reliant upon the discharges from the ensuing utilization of the biomass as a fuel. Expecting the carbon dioxide acclimatized to be transmitted on burning, the remaining emanations will rely upon the amount of energy of the biomass handling that might utilize petroleum products [135].

As it is known that inorganic compounds are also required in the nutrient media for algal cultivation. Nitrogen is chiefly used in the growth medium, so there is a possibility that by the use of microalgae, we can make it possible to remove the huge amount of nitrate compounds that are present in the wastewater and cause eutrophication [139].

The intrinsic advantage of microalgae is that they do not immediately compete with food by vying for a valuable agricultural area with established terrestrial crops. Accepting patterns for expanded strategy sustenance for transportation biofuels, microalgae as a feedstock can reduce more or less of the threats that first and second-age biofuels are causing to food security. Even though there is the most ideal probability for some microalgae strains as an additive in human weight control plans [135], it presently does not frame boundless dietary decisions. Cultivation of algae likewise decreases the contest for water assumed that it is ideally grown in wastewater [140], even though as recently referenced, the high supplement immersion can be significant to the practicality of its production for important results [141, 142].

In addition, where there is an emphasis to cultivate the feedstock away from the agronomic area, there is the benefit of using macroalgae and microalgae in cost reduction that is related to the scarcity of land resources. The cultivation of algae does not necessitate the same need for land as is needed for the land-dwelling biomass [143]. Generally, the cultivation of algae for the production of biofuels can most probably have the least effect on the security of food. It competes for the fuel versus food debate. The use of algal feedstock significantly reduces the stress on the first- and second-generation feedstock-related effects on the foodstuff and agronomical resources. Moreover, the diminished need for farmable land refutes the requirement for inescapable change of timberlands and forests. This lessens expected impacts on carbon sink and loss of biodiversity [144, 145].

Developing a biofuel industry based on algal cultivation can provide us with a lot of socio-economic advantages contributing to a publically maintainable result. Social sustainability includes, among further features, the possibility for an extra unbiased circulation of financial assistance through the public, comprising local and municipal societies, and enhancements in the life worth [146]. One of the most certain advantages is the fabrication of such an energy industry that can maintain and meet the long-term needs of the fuel along with more chances of employment. Such an industry can also be led to the growth of the economy in the local societies. Compared to this, the industries that are based on fossils are reliant on a limited number of resources [6]. As durable maintainable engineering, the production of biofuel using the microalgae can, moreover, offer openings for the progression of associated employments [147]. Industries based on the use of algae also provide chances for economical enhancement. Microalgae-centered manufacturers also provide a chance for economic growth in rural and topical areas [148]. Although the use of algae accounts for the higher costs of energy and production, still the production of algae-based biofuels prevents several limitations that were caused by the first and second-generation biofuels [124].

## Future prospects

Algal fuels might be superior to fossil fuels when considering the life-cycle evaluation, however, this field is still in its infancy. Despite the conflicting views, the concept of algae-based biofuels makes sense both philosophically and practically [39, 149–151]. Algal fuels manage a net positive energy recovery despite the currently underdeveloped manufacturing techniques, however, the precise amount is still up for debate. Algal biodiesel appears to have a reduced water footprint than biodiesel made from other crops [68, 69, 133, 149, 151]. Additionally, compared to open ponds, photobioreactors create an algal broth that is much more concentrated, which significantly lowers the dewatering expenses. It might be able to create dewatered algal biomass using tubular photobioreactors for about $4 per kilogram dry weight [152]. Moreover, there are probable enhancements in the cultivation and there is an aim to decrease the capital cost using the machinery of low cost for the further processing of algal biomass [117, 127]. Proper provisions of nutrients, CO_2_, and water in specific are supposed to be a limiting factor in the practicable cultivation of algae [153]. Another approach to significantly reducing the operational cost is to make possible the recycling of nutrients, water, and carbon dioxide during the production process [154]. Improvements are also required to effectively minimize the energy and cost required for the microalgae processing methods to be applicable at a commercial scale. By improving the techniques used at the harvesting stage, the costs associated with further processing steps to produce microalgae-based bioproducts and biofuels could be reduced [155].

Similar findings have been reached by additional independent research. As the production facility's scale is raised, the cost per unit of manufacturing algal biomass will further decline. The practicality of algae biofuels will probably be most impacted in the long run by genetic engineering. The possibilities for algal oil can be improved by improvements in methods for isolating the algae biomass from the water and extracting the oil from the biomass [150]. For instance, certain photosynthetic microorganisms' cells have been genetically modified to secrete oil that would typically be retained within the cell, making the process of recovering oil easier [156]. It will be a huge improvement if algal species can be developed to utilize atmospheric nitrogen instead of the nitrogen fertilizers that are currently needed. The manufacture of nitrogen fertilizers is highly dependent on petroleum [157].

Furthermore, putting in place the right regulatory frameworks to reflect the most affordable price can increase production viability as a long-term and sustainable replacement for fossil fuels. As evidenced by the comparatively rapid expansion of terrestrial feedstock, producers and consumers respond to the incentives provided by such policies (for example, in Brazil). Although the same regulations apply to the production of microalgae, the higher start-up costs and risks function as an additional barrier to investment compared to the less expensive agricultural-based production. Finding a policy mix that provides suitable incentives for third-generation biofuels while transitioning away from conventional approaches and managing the associated risks is likely to be as challenging given the technological advancements necessary to justify these incentives and the fuel's viability. Considering the potential of microalgae as a biofuel feedstock, accepting these challenges would appear to be founded on long-term optimism rather than utopian assumptions [152].

Moreover, recently more attention is being given to the co-culturing technique. Microalgae grow symbiotically with other heterotrophic microorganisms, including bacteria, yeast, fungi, and other algae/microalgae, in a co-cultivation method. They trade nutrients and metabolites, which boost productivity and make it easier to commercialize microalgal-based fuel. Co-cultivation makes it easier to gather biomass and value waste, which contributes to the development of an algae biorefinery platform for the generation of bioenergy [158].

Algae-based fuels seem quite promising. If the complete environmental impact of the latter forms of fuels is taken into account, they might already be seen as being competitive with petroleum-based fuels. We may be forced to abandon petroleum long before it runs out by climate change-related issues.

## Conclusions

The creation of third-generation biofuels, a superior type of biofuel, is the most promising application of the biomass obtained from algae species. Algae are adaptable plants that may flourish in a variety of aquatic environments, including water that contains a lot of salt or waste. Algae-producing facilities can be found in areas that are not suited for the growth of forests or agroecosystems. As a result, the production of algae does not compete with that of food, fiber, or fuel. Algae have been extensively exploited in industrial applications with the most intensive usage in the production of biofuels such as biobutanol, biodiesel, biohydrogen, or bioethanol. It has been reviewed that for the production of algal fuels, algae can be cultivated in all sorts of systems that can be closed, open, or hybrid. The production of biofuels depends upon several factors which influence the cultivation of algae. The practicality of algae biofuels will probably be most impacted in the long run by genetic engineering. Algal oil's prospects will be improved by improvements in methods for separating algal biomass from water and extracting oil from biomass. In the next 7–10 years, algae-based fuel production might be cost-effective, widely adaptable, and operational, but only if we continue improving our awareness of these magnificent species while also improving our capacity to tailor them for the specific aim of growing new energy industry. In the coming years, algae biomass could play a key role in resolving the conflict between food production and biofuel production.

## Data Availability

Not applicable.
